# Glomerular clusterin expression is increased in diabetic nephropathy and protects against oxidative stress-induced apoptosis in podocytes

**DOI:** 10.1038/s41598-020-71629-z

**Published:** 2020-09-10

**Authors:** Junling He, Kyra L. Dijkstra, Kim Bakker, Pascal Bus, Jan A. Bruijn, Marion Scharpfenecker, Hans J. Baelde

**Affiliations:** 1 Department of Pathology, Leiden University Medical Center, Leiden, The Netherlands; 2 Department of Pathology, Leiden University Medical Center, L1Q, room P0-107, P.O. Box 9600, 2300 RC Leiden, The Netherlands

**Keywords:** Diseases, Nephrology

## Abstract

Clusterin, a glycoprotein encoded by the *CLU* gene, is expressed in many tissues, including the kidney, and clusterin expression is upregulated in the glomeruli of patients with various forms of kidney disease. Here, we investigated the role of clusterin in diabetic nephropathy (DN). In this study, we found that glomerular clusterin expression was increased in both patients with DN and streptozotocin-induced diabetic mice and that it co-localised with the podocyte marker WT1, indicating clusterin is expressed in podocytes. In our in vitro analysis, we found no significant change in *CLU* mRNA expression in podocytes following stimulation with high glucose and angiotensin II; in contrast, *CLU* mRNA expression was significantly upregulated following methylglyoxal stimulation. Methylglyoxal treatment also significantly decreased the mRNA expression of the slit diaphragm markers *ZO-1* and *NEPH1* and significantly increased the mRNA expression of the oxidative stress marker *HO-1*. Lastly, we showed that pre-incubating podocytes with recombinant human clusterin protein increased podocyte survival, prevented slit diaphragm damage, and reduced oxidative stress‒induced apoptosis following methylglyoxal stimulation. Taken together, our results indicate that glomerular clusterin is upregulated in DN, and this increase in clusterin expression may protect against oxidative stress-induced apoptosis in podocytes, providing a possible new therapeutic target for DN and other kidney diseases.

## Introduction

Diabetic nephropathy (DN) is a major complication of diabetes and the leading cause of end-stage renal disease. Although the incidence of DN is increasing^1^, the mechanisms underlying the pathophysiology of DN are unclear, and no effective treatment for DN is currently available. However, one of the hallmarks of DN is a loss of podocytes, which are terminally differentiated and highly specialised epithelial cells in the glomerulus that serve as a critical component of the glomerular filtration barrier. Specifically, reduced numbers of podocytes are associated with the development of DN in patients with type 2 diabetes^2^, and changes in the structure and density of podocytes occur in the early stages of DN^3^. Moreover, Weil et al. reported that podocyte detachment is correlated with increasing albuminuria in these patients^4^. Therefore, new therapeutic strategies designed to prevent podocyte loss might slow or even prevent the progression of DN.

Clusterin (also known as Apolipoprotein J) is a disulphide-linked heterodimeric protein expressed in a wide range of tissues, including the kidney. Clusterin is a molecular chaperone that participates in a variety of biological processes, including lipid transport^5,6^, complement inhibition^7^, and regulation of apoptosis^8,9^. In addition, a number of studies have shown that clusterin can protect against oxidative stress. For example, Kim et al. reported that clusterin protects retinal pigment epithelial cells against oxidative stress via the PI3K/Akt pathway^10^. Besides, Jun et al. found that clusterin can protect cardiomyocytes from oxidative stress-induced apoptosis by inhibiting the Akt/GSK-3beta signalling pathway^11^, and Schwochau et al. found that clusterin protects cultured porcine proximal tubular cells against H_2_O_2_-induced damage^12^. In addition to playing a role in protecting against oxidative stress, biotinylated clusterin can bind to podocytes via the LDL receptor, thereby preventing complement-induced cellular damage in membranous glomerulonephritis (also known as membranous nephropathy)^13^. These findings collectively suggest that glomerular clusterin may protect podocytes from cellular damage.

Experimental studies also support the notion that clusterin may play a protective role in various forms of kidney disease. For example, reduced levels of clusterin have been associated with accelerated kidney fibrosis and aggravated tubular damage in a renal ischaemia–reperfusion injury model, thereby increasing kidney dysfunction^14,15^. Moreover, Jung et al*.* found that overexpressing clusterin reduces renal fibrosis in a unilateral ureteral obstruction model^16^, and Rosenberg et al. found that ageing clusterin-deficient mice develop moderate to severe mesangial lesions and have deposits of immune complex in the mesangium, compared to relatively few or no glomerular lesions in age-matched wild-type controls^17^. Finally, data from our group and others also suggest that clusterin may play a role in DN. Using microarray analysis, we found that glomerular *CLU* mRNA expression is significantly higher in two patients with DN compared to healthy individuals^18^. Moreover, using an autopsy cohort Nakatani et al. found that glomerular clusterin protein levels are 2.42-fold higher in ten diabetic patients compared to non-diabetic controls^19^. However, as these studies involved a relatively small number of patients, the role that increased glomerular clusterin expression plays in DN patients remains poorly understood.

To address this question, we examined glomerular clusterin expression in both a large cohort of patients with DN and a diabetic mouse model. In addition, we examined clusterin expression in a human podocyte cell line cultured under diabetic conditions. Finally, we examined whether treating podocytes with recombinant clusterin protein can protect the cells under diabetic conditions*.*

## Methods

### Human renal tissue

Two patient cohorts were used in this study. First, *CLU* mRNA was measured in microdissected glomeruli from a frozen biopsy cohort that included kidney tissue obtained from patients with DN (n = 24) and healthy subjects (n = 11); this cohort has been described previously^20^. Because no tissues were left from the biopsy cohort described above, we measured clusterin protein using a separate human biopsy cohort obtained from patients with histologically confirmed DN (n = 12); normal renal tissue samples obtained from healthy transplant donor kidney biopsies (n = 10) were used as a control group. The clinical parameters of these DN patients and healthy transplant donors can be founded in the Supplementary Table S1. The samples selected for this cohort were retrieved from the pathology archives of the Leiden University Medical Center (LUMC) in Leiden, the Netherlands, and were coded and treated anonymously in accordance with institutional guidelines and the code of conduct regarding the responsible use of human tissues from the Dutch Federa.

### Mouse model of type 1 diabetes

Diabetes was induced in 8-week-old female C57BL/6J mice (Harlan Laboratories, Indianapolis, IN) by three intraperitoneal injections of streptozotocin (STZ; 75 mg/kg body weight; Sigma-Aldrich, St Louis, MO) given at two-day intervals. Seven days after the first STZ injection, blood glucose levels were measured, and mice with a blood glucose level > 15 mmol/L were considered diabetic. Fifteen weeks after the induction of diabetes, diabetic mice (n = 5) were sacrificed, and the kidneys were harvested; age-matched control C57BL/6J mice (n = 5) were sacrificed at the same time points. The clinical biochemical parameters of these mice have been described previously^21^. All experiments were conducted in accordance with national guidelines regarding the care and use of experimental animals (DEC license 13163) approved by the Animal Experiments Committee (DEC) of the LUMC, the Netherlands.

### Immunohistochemistry

Paraffin-embedded human and mouse kidney tissues were sectioned at four µm thickness using a Leica microtome (Leica, Wetzlar, Germany), and the sections were subjected to heat-induced antigen retrieval using citrate (pH 6). Mouse kidney sections were stained with the following primary antibodies: rabbit anti-mouse clusterin (1:800, H-330, Santa Cruz Biotechnology, Santa Cruz, CA) or rabbit anti-mouse Wilms’ tumour protein (1:1,000, WT1; Santa Cruz Biotechnology); human kidney sections were stained with mouse anti-human clusterin (1:9,000, clone B-5, Santa Cruz Biotechnology). The anti-rabbit or anti-mouse Envision HRP-conjugated secondary antibody (Dako, Glostrup, Denmark) were used to visualise the primary antibodies, and diaminobenzidine (DAB + ; Dako) was used for chromogenic detection. The sections were visualised using a Philips Ultra-Fast Scanner 1.6 RA (Philips, the Netherlands) for immunohistochemical analysis.

### Glomerular clusterin immunohistochemistry staining score

A semi-quantitative score was used to analyse glomerular clusterin protein expression in the human kidney tissues. Specifically, glomerular clusterin expression was scored as 1, 2, or 3, corresponding to negative staining, weak staining, and moderate/strong staining, respectively. All glomeruli included in the human kidney biopsies were scored, and a mean was calculated. Glomerular clusterin protein expression scores can be found in the Supplementary Table S1.

Quantitative measurements were used to measure glomerular clusterin expression in the mouse kidney tissues. A total of 25 glomeruli per sample were randomly selected and measured quantitatively using ImageJ software (National Institutes of Health, Bethesda, MD).

### Cell culture

Conditionally immortalised human podocytes^22^, kindly provided by Moin Saleem (Bristol, UK), were cultured in RPMI 1640 medium (Gibco, Paisley, Scotland) supplemented with 10% foetal bovine serum (Sigma-Aldrich), 1% Insulin-Transferrin-Selenium (Invitrogen, the Netherlands), and 1% penicillin/streptomycin (Sigma-Aldrich). Podocytes were cultured at 33 °C (5% CO_2_) for proliferation and were differentiated by culturing in non-permissive conditions at 37 °C (5% CO_2_). Podocytes were used for experiments after 14 days of differentiation at 37 °C (5% CO_2_). All cells used in this study tested negative for mycoplasma infection.

### Incubation of podocytes with various compounds

After starving the cells in serum-free RPMI 1640 medium for 24 h, differentiated podocytes were treated as follows: puromycin aminonucleoside (PAN; 30 µg/ml) for 24, 48, or 72 h; normal glucose (5 mM) or high glucose (25 mM) for 24, 48, or 72 h; angiotensin II (1 µM) for 24, 48, or 72 h; methylglyoxal (0, 0.5, 1.0, or 1.5 mM) for 24 h; or *N*-acetyl-l-cysteine (NAC, 150 µmol/L) for 24 h. All compounds were obtained from Sigma-Aldrich, and all experiments were performed in triplicate.

### Quantitative real-time PCR analysis

RNA was extracted and analysed using quantitative real-time PCR (qPCR) as previously described^21^ using the following forward (FW) and reverse (RV) primers. The sequences of the primers used for the qPCR analysis can be found in Supplementary Table S2. PCR products were confirmed by sequencing. The *CLU, TJP1/ZO-1, NEPH1, HMOX1/HO-1, BAX,* and *BCL2* mRNA levels were normalised to the housekeeping gene *HPRT1*.

### Cell viability assay

Fully differentiated human podocytes were seeded in 96-well plates (5,000 cells per well), with three wells per condition. After starving in serum-free RPMI 1640 medium for 24 h, the podocytes were incubated with Milli-Q water or 1.5 mM methylglyoxal for 24 h either with or without pre-incubation for 4 h with (0, 0.25, 0.5, 1.0, or 2.0 µg/mL) recombinant human clusterin protein dissolved in PBS (R&D Systems, the Netherlands). Twenty-four hours after the addition of methylglyoxal, the medium was replaced with PrestoBlue Cell Viability Reagent (Invitrogen); 1 h later, fluorescence (570 nm) was measured using a Victor Multilabel plate reader (PerkinElmer, Waltham, MA). All experiments were performed in triplicate.

### Caspase 3/7 assay

Fully differentiated human podocytes were seeded in white 96-well plates. After starving in serum-free RPMI 1640 medium for 24 h, the podocytes were incubated with Milli-Q water or 1.5 mM methylglyoxal for 12 h with or without pre-incubated with 2.0 µg/mL recombinant human clusterin protein for 4 h. Then, an equal volume of Caspase-Glo 3/7 reagent (Promega) was added to the samples, and the plate was left in the dark at room temperature for 1 h on a shaker. Luminescence was measured with a TECAN-Infinite 200PRO (Switzerland).

### DCFDA/H2DCFDA staining

Intracellular ROS generation was detected using a 2′,7′-dichlorofluorescein diacetate (DCFDA, also known as H2DCFDA) fluorescent probe (Abcam, the Netherlands). Podocytes were seeded on glass slides in 24-well plates. The podocytes were treated with Milli-Q water or 1.5 mM methylglyoxal for 2 h with or without pre-treated with 2.0 μg/mL recombinant human clusterin protein for 4 h, followed by incubating with DCFDA (10 μmol/L) at 37 °C for 30 min in the dark and then washed with DFCDA kit buffer. The cell fluorescence  (Ex/Em = 485/535nm) was observed under a Zeiss confocal microscope (Zeiss, Germany).

### MitoSOX red staining of mitochondrial superoxide

Podocytes seeded on glass slides were treated as described above, followed by staining with 2.5 µM MitoSOX Red (Thermo Fisher Scientific, Waltham, MA) for 10 min. After rinsing with cold phosphate-buffered saline, the podocytes were fixed with 4% PFA for 5 min, followed by ice-cold 100% methanol for 10 min. Images were then captured using a Zeiss confocal microscope with ZEN software (Zeiss, Germany).

### Statistical analysis

Data were analysed using SPSS, version 25 (IBM, Armonk, NY). Summary data are presented as the mean ± SD of three independent experiments. Differences between two groups were analysed using the Student’s *t*-test, and differences between more than two groups were analysed using the one-way ANOVA. Differences with a *P*-value < 0.05 were considered statistically significant.

## Results

### Glomerular clusterin expression is upregulated in patients with DN

**Figure 1:**
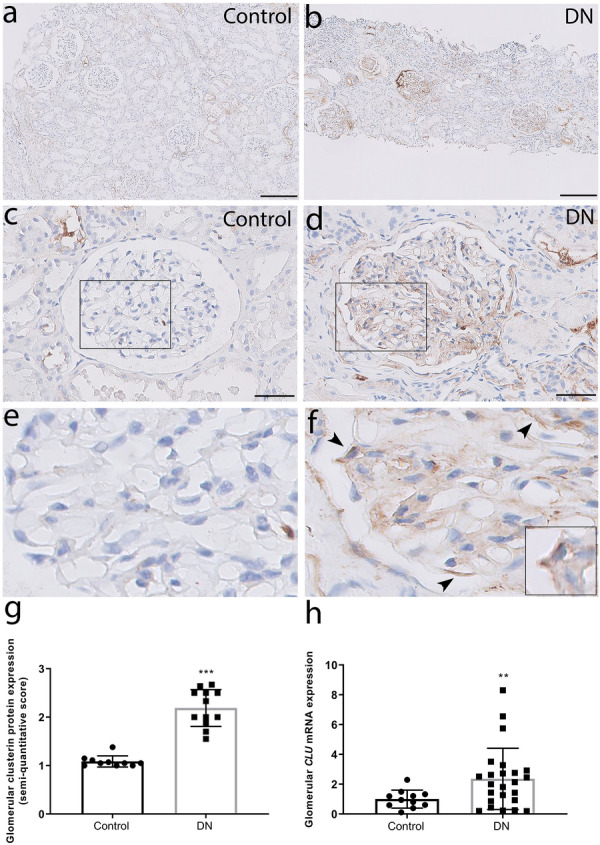
Glomerular clusterin expression is increased in patients with DN. (**a**,**b**) Representative images of kidney sections obtained from a healthy subject (Control; **a**) and a patient with DN (DN; **b**) stained for clusterin; the scale bars represent 200 µm. (**c**,**d**) Representative high-magnification images of a and b; the scale bars represent 50 µm. (**e**,**f**) The high-magnification views of the rectangles in (**c**,**d**). Clusterin staining was present along the outer side of the GBM at the place where the podocytes are located (arrowheads; the square shows a zoomed in image of a clusterin-positive podocyte). (**g**) Summary of clusterin staining (semi-quantitative score) in the glomeruli of healthy subjects (Control; n = 10) and patients with DN (DN; n = 12); ***P < 0.001 vs. control (Student’s t-test). (**h**) Summary of CLU mRNA measured in isolated glomeruli from healthy subjects (Control; n = 11) and patients with DN (DN; n = 24); **P < 0.01 vs. control (Student’s t-test).

First, we performed immunohistochemistry to examine kidney biopsies obtained from patients with DN and healthy kidney transplant donors and found little or no clusterin protein in the glomeruli of healthy subjects (Fig. 1a,c,e). In contrast, robust clusterin staining was present in the mesangial area and in podocytes in the kidney sections of DN patients (Fig. 1b,d,f, and Supplementary Fig. S1). Quantitative analysis revealed that glomerular clusterin protein expression was significantly higher in DN patient samples compared to controls (Fig. 1g). These results were supported by analysing *CLU* mRNA expression in another cohort, which showed significantly higher levels of *CLU* mRNA in the glomeruli of DN patient samples compared to controls (Fig. 1h).

### Glomerular clusterin expression is increased in a mouse model of type 1 diabetes and co-localises with podocytes

**Figure 2:**
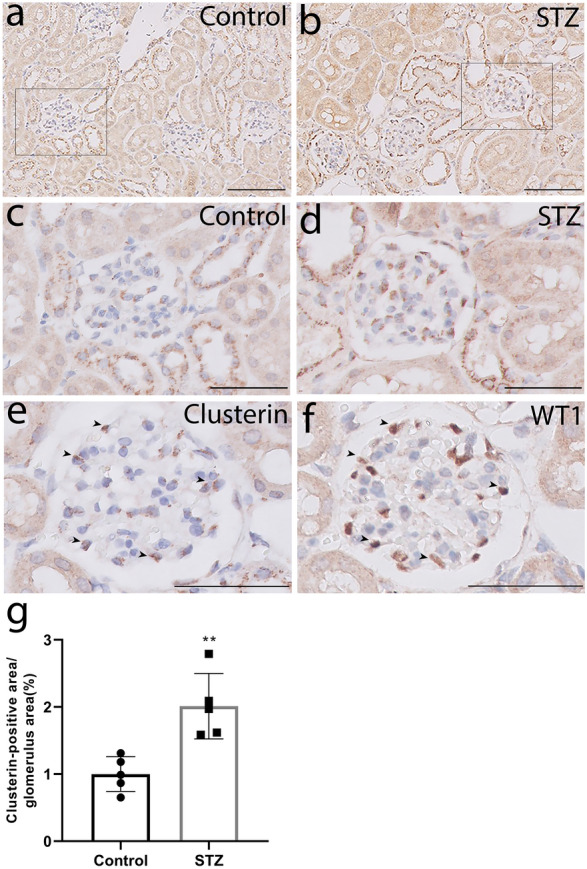
Clusterin expression is increased in the glomeruli of mice with type 1 diabetes and co-localises with podocytes. (**a**, **b**) Representative images of mouse kidney sections obtained from control mice (Control; **a**) and STZ-treated mice 15 weeks after diabetes was induced (STZ; **b**); the scale bars represent 100 µm. (**c**, **d**) The high-magnification views of the rectangles in (**a, b**); the scale bars represent 50 µm. (**e**, **f**) Representative images of the adjacent sections stained for clusterin (**e**) and WT1 (**f**), showing co-localisation of clusterin with WT1 in the glomeruli (arrowheads). (**g**) Averaged clusterin-positive area (measured by using ImageJ) in the glomeruli of control and STZ-treated mice 15 weeks after diabetes was induced; **P < 0.01 vs. control (Student’s t-test).

Next, we used immunohistochemistry to measure clusterin protein in the glomeruli of mice with STZ-induced type 1 diabetes and age-matched control mice. Clusterin staining was visible in the mouse glomeruli as a granular pattern (Fig. 2a–d). Compared to control mice, the STZ-treated mice had significantly more glomerular clusterin staining (Fig. 2g). Furthermore, we stained adjacent sections for clusterin and the podocyte marker WT1 and found that clusterin co-localised with WT1, confirming that clusterin is expressed specifically in podocytes (Fig. 2e,f).

### Altered expression of human clusterin in podocytes cultured under diabetic conditions

**Figure 3:**
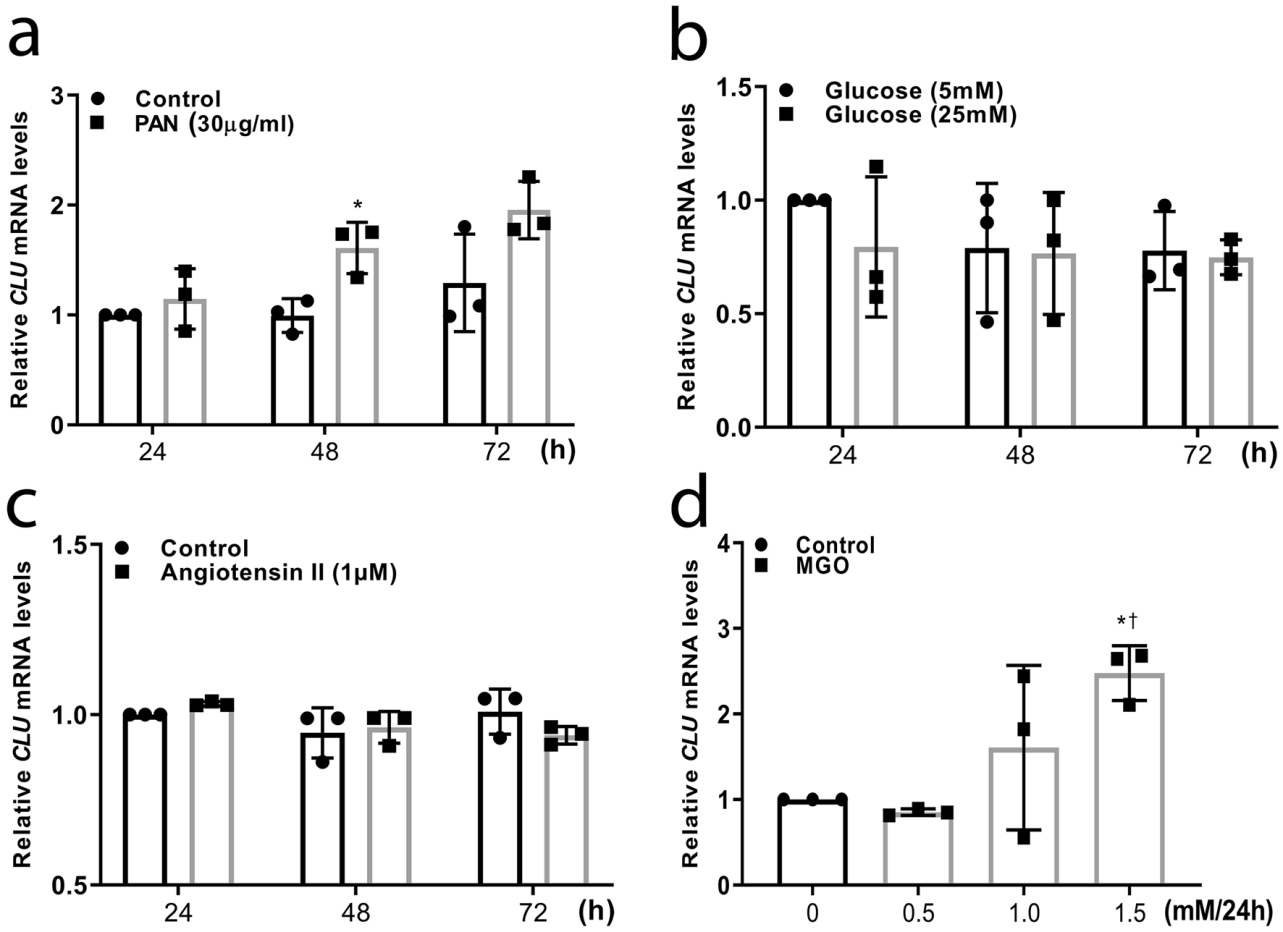
Clusterin expression in podocytes cultured under various diabetic conditions. (**a–d**) Human podocytes were cultured for the indicated times in PAN (30 µg/mL) (**a**), Glucose (5 mM or 25 mM) (**b**), Angiotensin II (1 µM) (**c**), or the indicated concentrations of MGO for 24 h (**d**). The *CLU* mRNA expression after these treatments was measured, the mRNA levels were normalised to the respective control. In (**a**–**c**), *P < 0.05 vs. the respective control at the same time point (Student’s t-test); in (**d**), *P < 0.05 vs. control, and †*P* < 0.05 vs. 0.5 mM MGO (one-way ANOVA). *PAN* puromycin aminonucleoside, *MGO* methylglyoxal.

Next, we investigated whether clusterin expression is altered in diabetes by measuring *CLU* mRNA in a human podocyte cell line under various diabetic conditions. First, we measured *CLU* mRNA in podocytes stimulated with puromycin aminonucleoside (PAN, 30 µg/mL), a toxic compound commonly used to induce podocyte cellular damage, and found significantly increased clusterin expression in stimulated cells (Fig. 3a). We then stimulated cells with either high glucose (Fig. 3b) or angiotensin II (Fig. 3c), but neither treatment significantly affected clusterin expression. Lastly, we stimulated podocytes with methylglyoxal, a major precursor in the formation of advanced glycated end products, which can increase ROS production and induce oxidative stress. Importantly, increased plasma levels of methylglyoxal have been reported in diabetic patients^23,24^. We found that culturing podocytes with 1.5 mM methylglyoxal for 24 h significantly increased clusterin expression compared to non-stimulated cells (Fig. 3d).

### Methylglyoxal increases oxidative stress and damages podocytes

**Figure 4:**
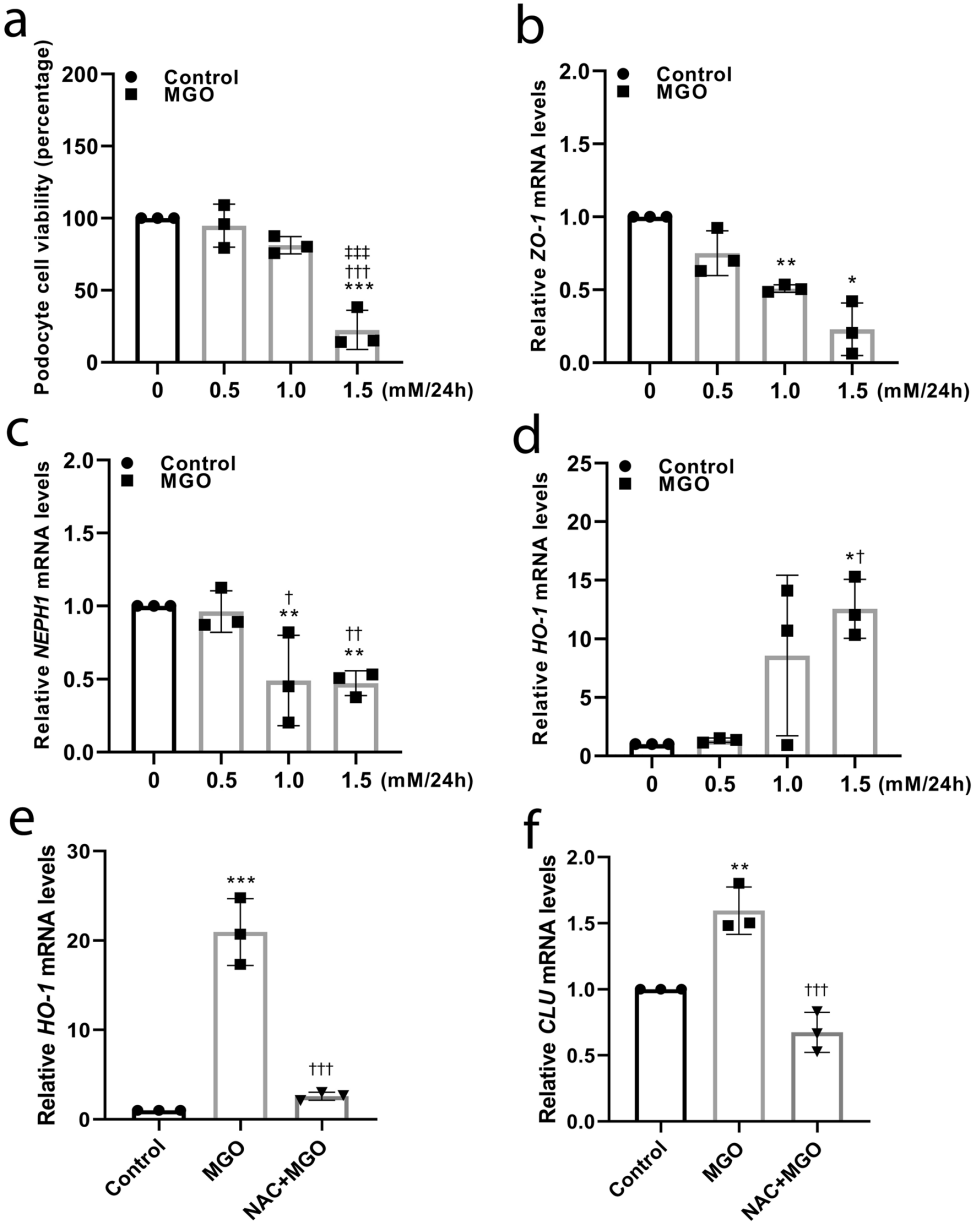
Methylglyoxal reduces podocyte viability, down-regulates ZO-1 and NEPH1 expression, and up-regulates HO-1 expression. (**a**) Cell viability was measured in podocytes incubated with the indicated concentrations of MGO for 24 h; ***P < 0.001 vs. control, ^†††^*P* < 0.001 vs. 0.5 mM MGO, and ^‡‡‡^*P* < 0.001 vs. 1.0 mM MGO (one-way ANOVA). (**b**–**d**) Podocytes were incubated with the indicated concentrations of MGO for 24 h, followed by measuring *ZO-1* (**b**), *NEPH1* (**c**), and *HO-1* (**d**) mRNA expression; the mRNA levels were normalised to the respective control; *P < 0.05 vs. control, **P < 0.01 vs. control, ^†^*P* < 0.05 vs. 0.5 mM MGO, and ^††^*P* < 0.01 vs. 0.5 mM MGO (one-way ANOVA). (**e**,**f**) Podocytes were incubated with MGO (1.5 mM) for 24 h, with or without pre-treatment with NAC (150 µmol/L) for 1 h, followed by measuring *HO-1* (**e**) and *CLU* (**f**) mRNA expression; the mRNA levels were normalised to the respective control; **P < 0.01 vs. control, ***P < 0.001 vs. control, and ^†††^*P* < 0.001 vs. MGO (one-way ANOVA). *MGO* methylglyoxal, *NAC N*-acetyl-l-cysteine.

To investigate the consequences of methylglyoxal stimulation, we measured cell viability in podocytes cultured for 24 h in increasing concentrations of methylglyoxal. We found that methylglyoxal reduced cell viability in a dose-dependent manner (Fig. 4a). Moreover, we found that methylglyoxal significantly decreased expression of tight junction protein 1 (*Tjp1/ZO-1*) and nephrin-like protein 1 (*NEPH1)*, both of which are involved in maintaining slit diaphragm integrity in podocytes (Fig. 4b,c). Finally, as discussed above, methylglyoxal can increase ROS production and cause oxidative stress. Consistent with this, we found that methylglyoxal significantly upregulated mRNA expression of Heme oxygenase 1 (HO-1), an oxidative stress marker (Fig. 4d). We also found that *N*-acetyl-l-cysteine (NAC), an anti-oxidant, can ameliorate the effect on HO-1 in methylglyoxal-treated podocytes (Fig. 4e). In addition, NAC was able to inhibit the methylglyoxal-induced increase of clusterin expression at the mRNA level (Fig. 4f). Taken together, these data suggest that oxidative stress might play an important role in up-regulating clusterin expression in podocytes under diabetic conditions.

### Clusterin protects against methylglyoxal-induced apoptosis in podocytes by reducing oxidative stress

**Figure 5:**
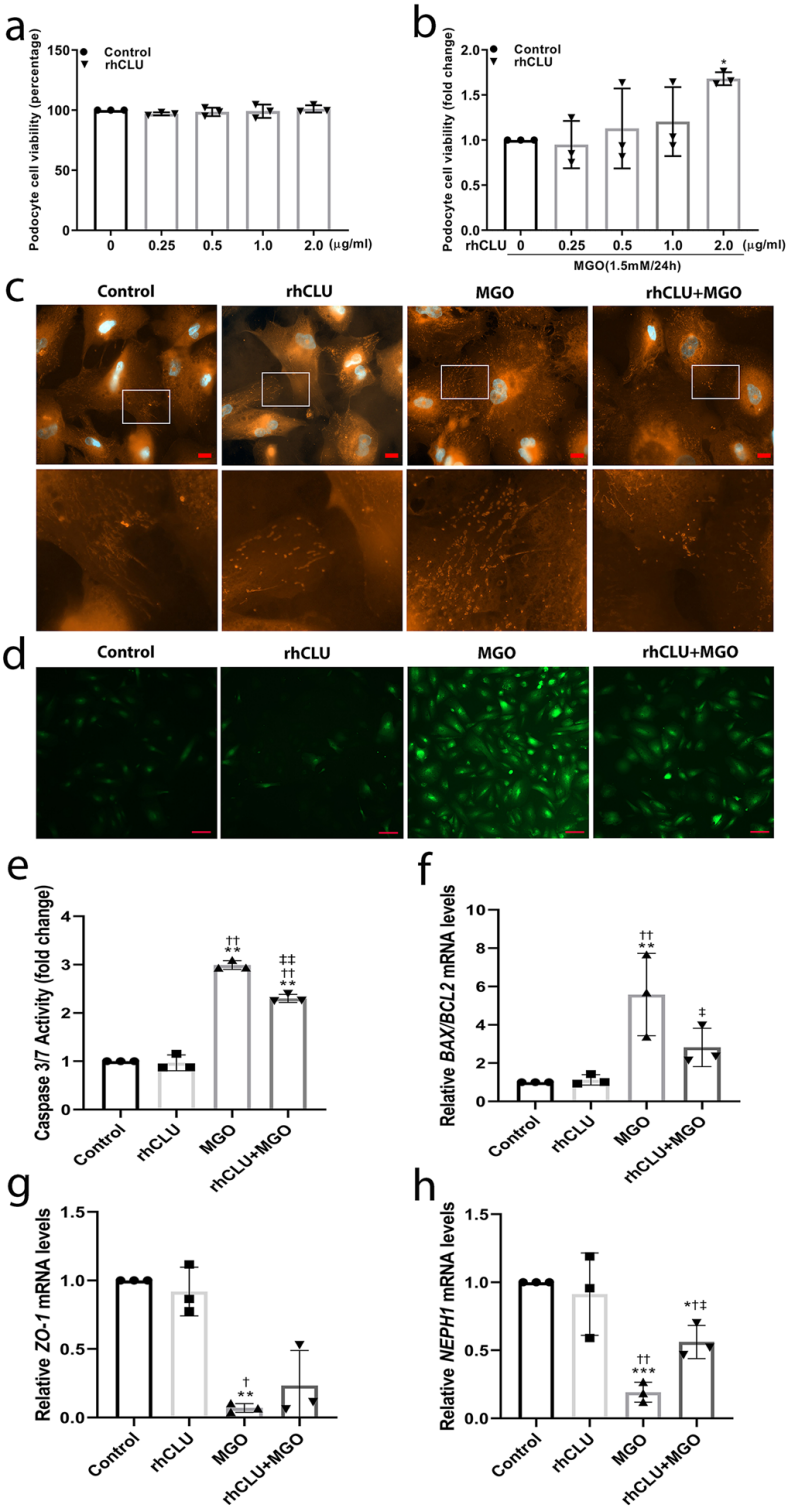
Recombinant human clusterin protein protects podocytes against methylglyoxal-induced oxidative stress and apoptosis. (**a**) Cell viability was measured in podocytes incubated with the indicated concentrations of rhCLU for 24 h. (**b**) Cell viability was measured in podocytes pre-incubated for 4 h with the indicated concentrations of rhCLU, followed by 24 h in the presence or absence of 1.5 mM MGO. Cell viability in the presence of MGO was normalised to cells stimulated in the absence of rhCLU; *P < 0.05 vs. 0 µg/mL rhCLU + 1.5 mM MGO (one-way ANOVA). (**c**) Representative images of podocytes treated with Milli-Q water (Control) or podocytes pre-treated for 4 h with 2.0 µg/mL rhCLU, 2 h with 1.5 mM MGO, or both, followed by MitoSOX Red staining. The scale bars indicate 20 µm and the boxed areas are shown at higher magnification in the bottom row. (**d**) Representative images of podocytes treated as described in (**c**), followed by DCFDA staining. The scale bars indicate 100 µm. (**e**) Caspase3/7 activity in podocytes treated with Milli-Q water (Control) or MGO (1.5 mM) for 12 h with or without pre-treated with 2.0 µg/mL rhCLU for 4 h. The measurement was normalised to the control; **P < 0.01 vs. control, ^††^*P* < 0.01 vs. rhCLU, and ^‡‡^*P* < 0.01 vs. MGO (one-way ANOVA). (f) Podocytes treated with Milli-Q water (Control) or MGO (1.5 mM) for 2 h with or without pre-treatment with 2.0 µg/mL rhCLU for 4 h, followed by measuring *BAX* and *BCL2* mRNA expression. The *BAX/BCL2* ratio was calculated and normalised to the control; **P < 0.01 vs. control, ^††^*P* < 0.01 vs. rhCLU, and ^‡^*P* < 0.05 vs. MGO (one-way ANOVA). (**g**,**h**) Podocytes treated with Milli-Q water (Control) or MGO (1.5 mM) for 2 h with or without pre-treated with 2.0 µg/mL rhCLU for 4 h, followed by measuring *ZO-1* (**g**) and *NEPH1* (**h**) mRNA expression; the mRNA levels were normalised to the respective control; *P < 0.05 vs. control, **P < 0.01 vs. control ***P < 0.001 vs. control, ^†^*P* < 0.05 vs. rhCLU, ^††^*P* < 0.01 vs. rhCLU, and ^‡^*P* < 0.05 vs. MGO (one-way ANOVA). *MGO* methylglyoxal, *rhCLU* recombinant human clusterin protein.

Next, we examined whether clusterin can protect podocytes during methylglyoxal stimulation. We therefore added recombinant human clusterin protein to podocytes and measured cell viability. We found that although culturing podocytes with recombinant human clusterin protein for 24 h had no effect on cell viability (Fig. 5a), pre-incubating podocytes with recombinant human clusterin protein (2.0 µg/mL) for 4 h significantly reduced methylglyoxal-induced cell death (Fig. 5b).

We then stained podocytes with MitoSOX Red to measure mitochondrial superoxide in order to test whether incubating cells with recombinant human clusterin protein reduced methylglyoxal-induced oxidative stress (Fig. 5c). We found that stimulating podocytes with 1.5 mM methylglyoxal for 2 h caused a substantial increase in MitoSOX Red staining in the cell cytoplasm, which was considerably reduced by pre-incubating cells with recombinant human clusterin protein (2.0 µg/mL). We also found that control podocytes and podocytes which were treated with rhCLU show a weak DCFDA staining. This staining was increased after methylglyoxal (1.5 mM) stimulation for 2 h. Pre-treatment with recombinant human clusterin protein (2.0 ug/mL) for 4 h considerably reduced the DCFDA staining in methylglyoxal-treated podocytes (Fig. 5d). These data suggest that clusterin can reduce methylglyoxal-induced oxidative stress in podocytes.

Furthermore, we examined whether methylglyoxal-induced cell death is mediated by apoptosis and whether clusterin can reduce apoptotic signalling in podocytes. We found an increased caspase 3/7 activity in methylglyoxal-treated podocytes compared to the control podocytes. Pre-incubation with recombinant human clusterin protein (2.0 µg/mL) for 4 h reduced the caspase 3/7 activity in methylglyoxal-treated podocytes compared to the podocytes treated with methylglyoxal only (Fig. 5e). Bax and Bcl-2 (encoded by the *BAX* and *BCL2* genes, respectively) are two major members of the Bcl-2 family and play a vital role in the apoptotic signalling pathway, as the balance between pro-apoptotic Bax and anti-apoptotic Bcl-2 determines the cells’ susceptibility to apoptosis^25^. We therefore measured the ratio between *BAX* mRNA and *BCL2* mRNA, and found that this ratio was significantly increased in methylglyoxal-stimulated podocytes; moreover, pre-incubating cells with recombinant human clusterin protein prevented this increase (Fig. 5f). These data indicate that clusterin prevents methylglyoxal-induced apoptosis in podocytes by reducing oxidative stress. Lastly, and consistent with our previous results, we found that pre-incubating podocytes with recombinant human clusterin protein for 4 h prevented the methylglyoxal (1.5 mM, 2 h)-induced downregulation of both *ZO-1* (Fig. 5g) and *NEPH1* (Fig. 5h).

## Discussion

Here, we report that glomerular clusterin expression was increased in patients with DN and a mouse model of STZ-induced diabetes. Moreover, we found that methylglyoxal significantly increased clusterin expression in cultured human podocytes, and that pre-treating podocytes with recombinant human clusterin protein protected against methylglyoxal-induced oxidative stress and apoptosis.

Urinary clusterin (uCLU) levels have been used as a biomarker for detecting kidney damage^26^. For example, Kim et al*.* recently reported that uCLU levels are significantly increased in patients with type 2 diabetes compared to non-diabetic subjects; the authors also found that uCLU is associated with the annual decline in estimated glomerular filtration rate (eGFR) and the progression of DN stage in patients with type 2 diabetes^27^. Moreover, Zeng et al*.* performed a consecutive cohort study and found that uCLU levels are higher in type 2 diabetic patients with DN compared to control subjects, and that this increase is positively correlated with the urinary albumin-creatinine ratio^28^. Interestingly, studies suggest that changes in uCLU levels may be due—at least in part—to changes in renal *CLU* expression. For example, Nakatani et al. performed a proteomics analysis in an autopsy cohort and found that clusterin protein is significantly increased in the glomeruli of patients with DN^19^. Similarly, using microarray analysis, we previously found that *CLU* mRNA expression is significantly increased in isolated glomeruli of two patients with DN compared to healthy individuals^18^. In the present study, we found that *CLU* mRNA expression was increased in micro-dissected glomeruli of DN patients, and we found increased clusterin protein expression in the mesangium and podocytes in DN patient biopsies. Importantly, this cohort consists of kidney samples taken from patients with DN ranging from class 2 to class 4; thus, a larger biopsy cohort would be helpful in determining whether clusterin expression is correlated with the class and/or severity of DN.

To support our findings in patients with DN, we also measured glomerular clusterin protein expression in a mouse model of type 1 diabetes and observed increased glomerular clusterin protein expression in mice with STZ-induced type 1 diabetes compared to control mice. This finding is consistent with a previous report by Tunçdemir et al*.* showing increased glomerular clusterin expression in a rat model of type 1 diabetes^29^. Our finding that clusterin co-localised with the podocyte marker WT1 in mice is also consistent with our clusterin immunostaining results in renal biopsies obtained from patients with DN, indicating that podocytes are the clusterin-expressing cell type in glomeruli, which is consistent with reports that clusterin expression is upregulated in various epithelial cells during stress^30,31^.

The clusterin protein is post-translationally modified to produce a secreted isoform and a nuclear isoform, which play opposite roles in apoptosis^8^. Our mouse data show that the clusterin staining in the glomeruli of diabetic mice had a granular pattern, which suggests that this is the secreted, anti-apoptotic isoform. Several experimental studies support the notion that clusterin has protective properties in various kidney diseases. For example, clusterin deficiency accelerates renal fibrosis and increases renal inflammation in a mouse model of renal ischaemia–reperfusion injury^15^. Moreover, overexpressing clusterin reduces renal fibrosis in the mouse unilateral ureteral obstruction model^16^. Rosenberg et al*.* found that aged clusterin-deficient mice, but not aged control mice, have moderate to severe mesangial matrix expansion^17^. In addition, Ghiggeri et al. illustrated that low levels of clusterin in serum negatively affect the clinical outcome in patients with nephrotic syndrome^32^. However, whether clusterin plays a protective role in DN remains elusive.

It is still controversial whether glomerular clusterin is derived from the circulation or synthesized by resident glomerular cells. Yamada et al. found that clusterin is upregulated in glomerular mesangial cells during the course of immune-mediated injuries^33^. Laping et al*.* demonstrated that thrombin increases clusterin expression in mesangial cells and glomerular epithelial cells at the mRNA level^34^. In the present study, we focussed on the podocytes according to our findings from human biopsies and mice tissue. In in vitro experiments using a human podocyte cell line, we found that PAN, a compound commonly used to induce cell damage in podocytes, significantly increased *CLU* mRNA expression. In contrast, exposing cells to either high glucose or angiotensin II had no significant effect on clusterin expression. However, we demonstrate that treating podocytes with methylglyoxal to mimic diabetes-related oxidative stress increased *CLU* mRNA expression. Methylglyoxal is a major precursor in the formation of advanced glycated end products, which can increase ROS production and cause oxidative stress. Interestingly, studies have shown elevated levels of methylglyoxal in diabetic patients^23,24^. Moreover, Kim et al*.* reported that methylglyoxal-induced oxidative stress might play a role in podocyte apoptosis in DN^35^. Here, we found that stimulating podocytes with methylglyoxal for 24 h increased *CLU* mRNA expression, as well as expression of the oxidative stress marker (HO-1) at the mRNA level. We did not find a difference between *HO-1* mRNA expression in control podocytes and in angiotensin II-treated podocytes with the concentration we used (data not shown). Furthermore, we show that methylglyoxal treatment increased mitochondrial superoxide and intracellular ROS regeneration in podocytes. In addition, we found that NAC, an anti-oxidant, ameliorated the effect on HO-1 in methylglyoxal-treated podocytes and inhibited the methylglyoxal-induced increase of clusterin expression at the mRNA level. Taken together, these data suggest that oxidative stress, but not hyperglycemia or the renin-angiotensin system, is the principal factor underlying the upregulation of clusterin expression in podocytes under diabetic conditions.

Our findings also show that pre-treating podocytes with recombinant clusterin protein can help to protect podocytes against methylglyoxal-induced apoptosis. We observed that pre-incubating podocytes with recombinant human clusterin protein (2.0 µg/mL) for 4 h significantly reduced methylglyoxal-induced cell death. Pre-incubating podocytes with recombinant human clusterin protein prevented an increase of the *BAX/BCL2* ratio, as well as of caspase 3/7 activity. The protective function of clusterin may have clinical relevance. Evidence suggesting how clusterin might exert this protective role comes from several in vitro studies. For example, Rastaldi et al*.* found that biotinylated clusterin can bind to podocytes via the LDL receptor and can protect podocytes from complement-induced injury in membranous glomerulonephritis^13^. Kim et al*.* found that clusterin can protect human retinal pigment epithelial cells from oxidative stress via the PI3K/Akt pathway^10^, and Jun et al*.* showed that clusterin protects cardiomyocytes from oxidative stress-induced apoptosis by inhibiting the Akt/GSK-3beta signalling pathway^11^. Yu et al*.* demonstrated that *p*-Akt expression is upregulated in mesenchymal stem cells following pre-treatment with clusterin, and that the Akt inhibitor LY29440236 partially abrogated the protective function of clusterin^36^. Therefore, clusterin may modulate Akt-related pathways to slow or even halt the progression of DN. The mechanism of how cytoprotection is mediated by clusterin in the different pathways needs to be further elucidated. Further in vivo studies are also needed to confirm our hypothesis that clusterin may slow or even halt the progression of DN.

In summary, we provide evidence that glomerular clusterin is upregulated in DN and that experimentally-induced oxidative stress up-regulates clusterin expression in podocytes. Moreover, pre-treating podocytes with recombinant human clusterin protein protects against apoptosis by reducing oxidative stress-induced under diabetic conditions, suggesting a possible therapeutic target for treating DN.

## Supplementary information

Supplementary Information
