# Supplementary material for: Glomerular clusterin expression is increased in diabetic nephropathy and protects against oxidative stress-induced apoptosis in podocytes
Source: Sci Rep. 2020 Sep 10;10:14888. doi: 10.1038/s41598-020-71629-z (PMC7484791; doi:10.1038/s41598-020-71629-z)
Supplement: Supplementary file 1 — Supplementary Information [file 41598_2020_71629_MOESM1_ESM.docx]

**Glomerular clusterin expression is increased in diabetic nephropathy and protects against oxidative stress‒induced apoptosis in podocytes**

Junling He^1*^, Kyra L. Dijkstra^1^, Kim Bakker^1^, Pascal Bus^1^, Jan A. Bruijn^1^, Marion Scharpfenecker^1^, Hans J. Baelde^1^

1. Department of Pathology, Leiden University Medical Center, Leiden, The Netherlands

Running title: Clusterin in diabetic nephropathy

* Corresponding author: Junling He ( [j.he@lumc.nl](about:blank) )

**Supplementary Table 1. Clinical parameters of DN patients and healthy subjects**

Abbreviations: DM: diabetes mellitus; DN: diabetic nephropathy; HTN: hypertension; HbA1c: glycated haemoglobin.

**Supplementary Figure 1.**


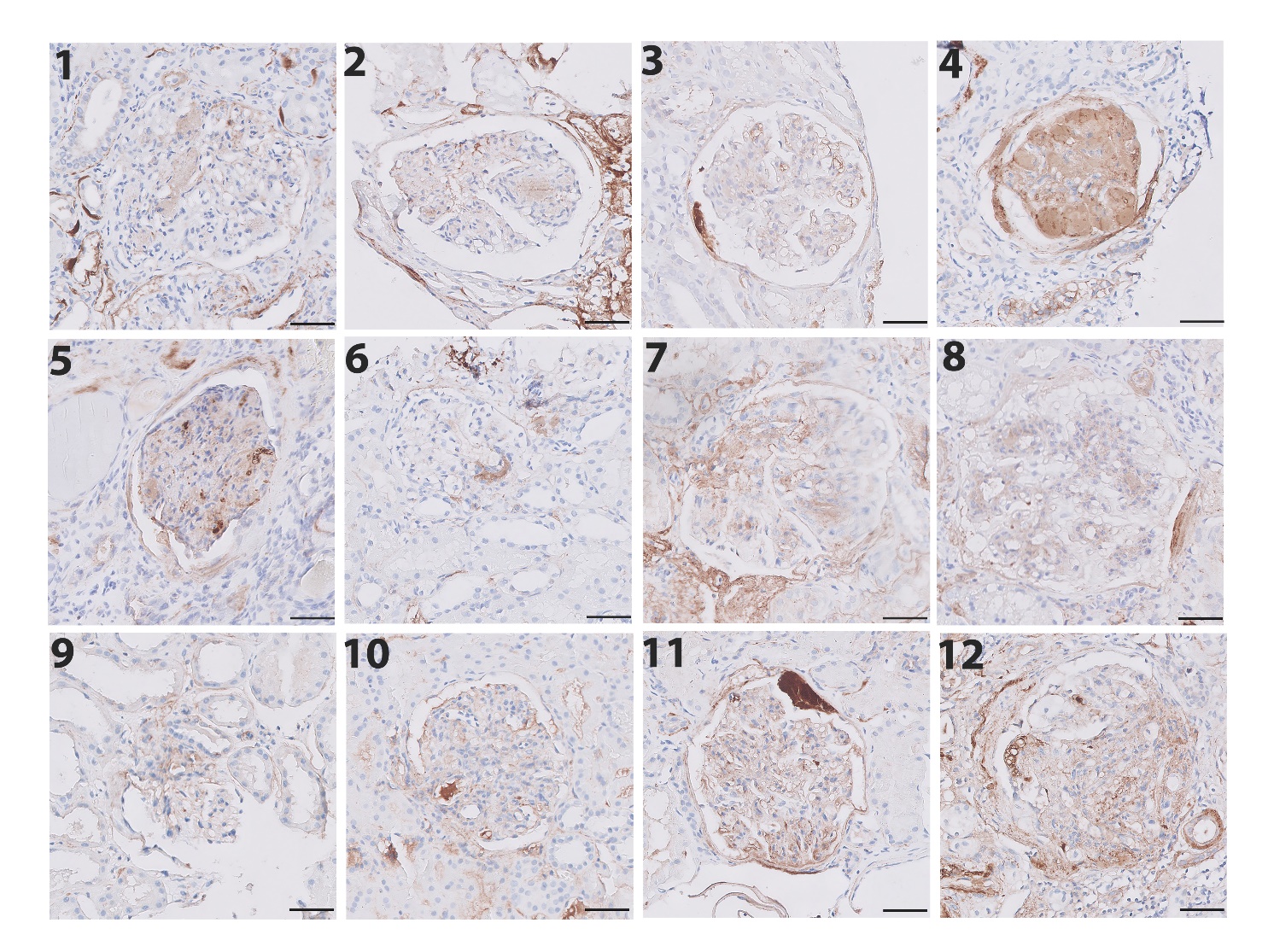


**Supplementary Figure 1.** **Clusterin immunohistochemistry on biopsies from patients with diabetic nephropathy**

Representative pictures of clusterin staining on paraffin-embedded sections of the biopsies of 12 individual patients with diabetic nephropathy; the scale bars represent 50 µm.

**Supplementary Table 2. Primer sequences used for the qPCR analysis**

| **Gene name** | **Forward (5’-3’)** | **Reverse (5’-3’)** |
| --- | --- | --- |
| *CLU* (human) | AATGCTGTCAACGGGGTGAA | TGGGAGCTCCTTCAGCTTTG |
| *TJP1/ZO-1* (human) | GAACGAGGCATCATCCCTAA | CCAGCTTCTCGAAGAACCAC |
| *NEPH1* (human) | GAATAAGACACCTCCTCCTG | GACTGCTTAGGAGAAGAGAG |
| *HMOX1/HO-1*(human) | CCGATGGGTCCTTACACTCAG | AAAGTTCATGGCCCTGGGAG |
| *BAX* (human) | TGCTTCAGGGTTTCATCCAG | GGCGGCAATCATCCTCTG |
| *BCL2* (human) | AGGAAGTGAACATTTCGGTGAC | GCTCAGTTCCAGGACCAGGC |
| *HPRT1* (human) | AGATGGTCAAGGTCGCAAGC | TCAAGGGCATATCCTACAACAAAC |

|  |
| --- |
